# Mammography as a diagnostic tool in women aged 35 to 39 presenting to the symptomatic breast clinic

**DOI:** 10.1186/bcr2997

**Published:** 2011-11-04

**Authors:** J Long, CE Richardson, J Barrett, C Deacon, P Matey

**Affiliations:** 1 The Royal Wolverhampton Hospitals NHS Trust, Wolverhampton, UK

## Introduction

Recent guidelines advise that mammography should only be offered to women between the ages of 35 and 39 if clinical examination raises concern or a targeted ultrasound of the breast reveals a suspicious abnormality [1]. The aim of this study was to assess the role of mammography as a diagnostic tool in women aged 35 to 39 presenting to the symptomatic breast clinic.

## Methods

All women aged between 35 and 39 years presenting to the symptomatic breast clinic between April 2010 and March 2011 were eligible for inclusion. Data were collected on demographics, presenting symptoms, radiological investigation and diagnosis.

## Results

A total 112 of 124 women identified had mammography performed as part of their initial assessment; one woman presented with a solid lump which was indeterminate on ultrasound but proved to be breast cancer on biopsy. The majority of patients were diagnosed with mastalgia, benign nodularity or fibroademona. Core biopsies confirmed benign pathology in eight women. Of the 12 women who did not have mammography, benign pathology was diagnosed using clinical examination and ultrasound, with breast cysts and sepsis being the commonest causes of pathology.

## Conclusion

The role of mammography in women aged 35 to 39 presenting to the symptomatic breast clinic should be reassessed. Our data support the guidelines that ultrasound is the imaging method of choice in this age group.
